# Fallow deer approaching humans are more likely to be seropositive for *Toxoplasma gondii*

**DOI:** 10.1098/rsos.250159

**Published:** 2025-08-13

**Authors:** Andrew R. Ryan, Annetta Zintl, Laura L. Griffin, Amy Haigh, Matthew Quinn, Pietro Sabbatini, Bawan Amin, Simone Ciuti

**Affiliations:** ^1^ Laboratory of Wildlife Ecology and Behaviour, University College Dublin School of Biology and Environmental Science, Dublin, Leinster, Ireland; ^2^ University College Dublin School of Veterinary Medicine, Dublin, Leinster, Ireland; ^3^ Department of Forest Resources Management, The University of British Columbia Faculty of Forestry, Vancouver, British Columbia, Canada; ^4^ Department of Biosciences, Durham University, Durham, UK

**Keywords:** animal behaviour, toxoplasmosis, *Toxoplasma gondii*, risk taking, host manipulation, *Dama dama*, parasitology, serology

## Abstract

*Toxoplasma gondii* infection has been linked to dampening hosts anti-predator behaviour particularly in laboratory conditions with rodents. Little is known about the role of *T. gondii* within more complex ecological contexts involving large mammals. Therefore, we aimed to determine the prevalence of *T. gondii* infection in a population of free-living fallow deer (*Dama dama*). In addition, we assessed whether there was a link between deer seropositivity and space use where deer may be more likely to be exposed to *T. gondii* (e.g. closer to human infrastructure). Finally, we determined whether infection with *T. gondii* was linked to deer risk-taking behaviour. To achieve our goals, we estimated seropositivity and combined it with spatial distribution and behavioural data of individually recognizable deer ranging from those that avoid humans (risk avoiders) to those who beg for food (risk takers). We found *T. gondii* to be quite widespread in this population with a seropositivity rate of approximately 20%. We found no correlation between *T. gondii* seropositivity and space use in the park, therefore we were unable to determine how the deer were exposed. We did however find that seropositive deer were also more likely to take risks, opening new avenues to explore *T. gondii*’s dynamics in the wild.

## 1. Introduction

It has been argued for some time that some trophically transmitted parasites (where the definitive host becomes infected by predation or scavenging on an intermediate host) have evolved the ability to manipulate their hosts’ behaviour to increase the likelihood to complete their life cycle [1]. One of the first parasites for which the host manipulation hypothesis was suggested was *Toxoplasma gondii* [2]. *T. gondii* infects feline species as its only definitive hosts and most, if not all, warm-blooded animals as intermediate hosts [3]. Sexual reproduction in the epithelial cells of the feline small intestine results in the production of millions of oocysts which are released in the cat’s faeces [4]. Under cool, moist conditions, oocysts can survive in the environment for months or even years [5]. Once they are ingested by an intermediate host, the oocysts give rise to tachyzoites which invade various organs and tissues undergoing repeated rounds of asexual reproduction [6]. Eventually, the immune response of the intermediate host causes the tachyzoites to convert into less metabolically active bradyzoites, which encyst in muscle and nervous tissues [6]. The cycle is completed if and when the intermediate host is consumed by another feline definitive host.

It has been suggested that *T. gondii* may manipulate its intermediate hosts’ behaviour—namely increased risk taking or a reduction in risk aversion—to make them more susceptible to predation, therefore increasing the likelihood that a definitive host eats the infected meat. Previous research has shown that the parasite may affect the synthesis of testosterone [7] and neurotransmitters such as dopamine [8]. Moreover, inflammatory responses to the presence of *T. gondii* cysts in brain tissue have also been associated with host behavioural changes [9]. The best-known example of the host manipulation hypothesis reported in rodents demonstrated that *T. gondii* infections reduced natural aversion to cat urine [10], increased reaction times [11] and decreased neophobia [2]. Tan & Vyas (2016) found that rats infected with *T. gondii* expressed higher levels of risk taking in a balloon analogous risk task. This experiment presented rats with a food tray and two levers. When pulled, one ‘addition’ lever added food to the tray and the other ‘cash out’ lever allowed the rat access to the food that had accumulated in the tray. The rats could pull the addition lever as many times as they wanted; however, after the first pull there was an increasing risk of losing all of the food in the tray on each subsequent pull, much like adding breaths of air to an overinflated balloon. As with most animals this experiment has been run on, rats were found to be relatively risk averse as they played conservatively by pulling the addition lever less than the theoretically optimal number of times before cashing out. Rats that were infected with *T. gondii* pulled the lever more times than there uninfected counterparts, however still below the optimum. A marked reduction in the natural aversion to leopard urine has also been observed captive chimpanzees [12].

Comparable studies on free-living animals are less common, mostly due to difficulties in collecting epidemiological, diagnostic and behavioural data in wild settings simultaneously requiring a multi-disciplinary approach. While a range of publications have reported data on *T. gondii* prevalence in various species of wildlife [13], fewer studies have examined potential behavioural changes [14–17]. More research is needed to disentangle the disease ecology and the role of *T. gondii* within more complex ecological contexts involving large mammals in the wild. To fill this gap, we conducted a study on a model population of fallow deer (*Dama dama*) living in one of the largest urban parks in Europe: Phoenix Park in Dublin, Ireland. The goals of our study were threefold. The first was to determine the seroprevalence of *T. gondii* infection in our model population, often interacting or spatially overlapping with humans and their pets because of the urban settings. The second was to determine whether there was a link between deer seropositivity and spatial proximity to buildings with domestic cats where deer would be more likely to become exposed to the parasite. Finally, we aimed to determine whether infection with *T. gondii* was linked to risk-taking behaviour in these deer, namely the likelihood to approach park visitors. We considered likelihood to approach visitors to be a good proxy for risk taking: for instance, playback experiments have found ungulates react as strongly to human voices as the calls of many of their natural predators [18]. In this particular population, the main causes of mortality are annual culls and collisions with vehicles making humans the main predator in the park [19]. However for many deer, the potential reward of highly calorific food offered by visitors is considered worth the risks [19]. To achieve our goals, we gathered unique data, which were collected adopting a multi-disciplinary approach involving behavioural and disease ecologists. We combined seropositivity information with spatial distributions and the behavioural data of individually recognizable deer, ranging from those that avoid humans (risk avoiders) to those who beg for food (risk takers) over multiple years.

## 2. Material and methods

### 2.1. Study site and population

The Phoenix Park is a 709 ha-walled park, situated within 2 km of Dublin city centre. Two of the main habitats in the park are grasslands and assorted woodlands. The park hosts an estimated 10 million visitors per year, creating the ideal location to examine human–wildlife interactions [19–21]. The herd consists of approximately 600 fallow deer, of which roughly 80% are identifiable via ear-tags that are applied each year to the majority of newborns during the fawning season [22]. As there are no natural predators capable of preying upon adult deer in the park, the population is managed by yearly culls. Culls were structured by age and sex classes to maintain a natural population structure with individuals selected at random to avoid artificial selection of behavioural traits.

Griffin *et al*. [19] have documented in detail the behaviour of the majority of the individually recognizable deer of this population. In particular, focusing on their level of engagement with park visitors and willingness to tolerate human presence and close contact interactions to obtain processed human food [19]. Most of the deer usually avoid human contact and maintain a distance greater than 50 m. A quarter of this population, however, tend to approach humans consistently and take advantage of additional artificial food sources [19].

As the definitive hosts for *T. gondii*, cats probably play an important role in disease dynamics in the park. As New Zealand has done substantial work on cat management when interacting with public spaces, we used definitions from the national ‘Code of welfare: Companion cats’ [23]. They draw distinctions between three types of cats that may occupy public spaces; companion cats, stray cats and feral cats. A companion cat ‘lives with humans as a companion and is dependent on humans for its welfare’, a stray cat is ‘a companion cat which is lost or abandoned and which is living as an individual or in a group (colony)’, and a feral cat ‘is not a stray cat and which has none of its needs provided by humans’. Stray cats will often have many of their needs indirectly supplied by humans and are likely to interbreed with the companion cat population. Feral cats, on the other hand, generally do not live around centres of human habitation and populations are largely independent on influx from companion cat populations [23].

Anecdotally, park rangers mention that a stray cat colony existed on the grounds of the campus of Ordnance Survey Ireland (OSI; the Irish state’s national mapping body) in the western quadrant of the park. However approximately 10 years before the start of this study the colony was culled, leaving one individual. This remaining individual was fed by the staff of the OSI throughout the study period. There were also infrequent reports of companion cats observed in the park. The perimeter of the park boarders onto housing estates and apartment blocks where most of these companion cats presumably lived. These cats probably did not travel too far into the park, possibly less than 100 m from their homes [24]. As the park is embedded in Dublin city, there is no chance of a population of truly feral cats existing in such an urban environment, by definition [23]. Any companion or stray cats likely clustered around artificial structures for shelter, with cats on the more stray end of the spectrum possibly preferring abandoned structures of which there are a handful around the park.

### 2.2. Collection of blood samples

Blood samples were collected during the fawning period in June 2020 and June 2021 during the routine activities in the park when neonate deer are trapped and ear-tagged [22]. Fallow deer adopt a ‘hider’ strategy meaning that neonates hide in the vegetation while waiting for periodic visits of the mother for lactation, in general, this behaviour lasts for 2−3 weeks after birth after which point the fawns join the herd [25]. Precise data on the point at which fawns join the herd in this population can be found in Amin *et al*. [26]. For tagging, fawns are captured by a team of 10−15 people sweeping through the understory vegetation across the fawning sites of the park. When a fawn is spotted, it is caught using fishing nets, handled and quickly released. Amin *et al*. [22] includes a full description of the capture, handling and tagging operations following the highest standards of wildlife handling and welfare. As part of this process, small clips from the fawns’ ears were taken for genetic sampling, in most cases producing one or few drops of blood. These small amounts of blood were collected from the clip site using Nobuto blood filter-paper strip (Advantec 800700) as described by Nobuto [27]. The filter paper was then placed in individual sterile test tubes and kept on ice for transfer into a freezer by the end of the day. All animal handling was conducted with permission from the UCD Animal Research Ethics Committee, under the permit AREC-E-18-28. The serostatus of the fawns was used to indirectly determine whether their mothers had antibodies against *T. gondii*. The identity of the mothers was determined via behavioural observations (e.g. suckling, grooming, following between the mother and the fawn) as described in Griffin *et al*. [19] and Amin *et al*. [26].

In addition, blood samples were collected during the annual culls of 2020 and 2021, in the period between November and January. The cull was conducted by professional stalkers hired by the government body responsible for the management of deer in the park, the Office of Public Work (OPW), under a hunting permit issued by the National Park and Wildlife Services (NPWS). During the cull, we collected blood samples in 50 ml falcon tube as the carcass was hung up. Sera were collected following centrifugation (2500*g* for 10 min) and stored at −20°C. In contrast to the fawning blood sample collection, where we indirectly sampled mothers corresponding to the fawns (thus, only females were indirectly sampled), we directly collected both male and female deer sera samples during the cull. The cull targeted individual deer at random following a precise culling plan with quotas indicating the minimum number of age and sex classes to be removed from the population. Griffin (personal communication), who was responsible for overseeing deer welfare during culling operations, quantified the proportion of begging individuals (*sensu* Griffin *et al*. [19]) in the culled and general population to be similar across years.

### 2.3. ELISA test: seropositivity in the sampled deer population

We choose to use serology to determine infection status as this method has been proven to work well to detect latent *T. gondii* infections [28]. Sera were tested for the presence of specific antibody using the ID Screen toxoplasmosis indirect multi-species enzyme-linked immunosorbent assay (ELISA) kit (product code: TOXOS-MS-2P). This specific kit is designed to give robust results between a wide range of animals including dogs, sheep, pig and ruminants. For the fawn samples collected on Nobuto blood filter-paper strips, sera were eluted from filter paper squares by soaking them overnight in the dilution buffer provided by the kit. Depending on the amount of blood that had been captured on the filter paper, samples consisted of 0.1, 0.5, 1 or 2 squares of dimensions 0.5 cm × 0.5 cm. Samples with two squares were eluted in 200 µl of dilution buffer, samples with one square were eluted in 150 µl of dilution buffer, samples with 0.5 squares were eluted in 100 µl of dilution buffer, respectively. All samples were applied directly to the test plate (i.e. analysed without further dilution) and each sample was run in duplicate.

The serum samples derived from the blood collected during the deer cull were analysed at a dilution of one-tenth as recommended by the kit manufacturer. Again, two replicates were tested for each sample. Positive and negative controls provided in the kit were included on each plate. For each sample, a sample/positive percentage (*S*/*P*%) was calculated from the optical density (OD) values using the following formula:


$$S/P\%=\frac{OD\left[sample\right]-OD\left[negative\;control\right]}{OD\left[positive\;control\right]-OD\left[negative\;control\right]}\times 100.$$

According to the kit manufactures, *S*/*P*% values less than 40 are considered negative, *S*/*P*% values from 40 to 50 are equivocal and *S*/*P*% values greater than 50 are positive. In order to determine whether slight differences in the blood sample on the filter paper and the volume of elution buffer would affect the outcome of the analysis, a panel of 11 deer sera were analysed both at dilutions of 1/2 and 1/10. While *S*/*P*% values increased somewhat at the lower dilution factor, the test outcome was mostly unaffected (results in electronic supplementary material, S1).

### 2.4. Analysis of space use by deer in the park with respect to buildings

The space use of individual deer was determined using a survey carried out during daylight hours on a weekly or biweekly basis from September 2018 to December 2021. Surveys tracked the spatial location of individually recognizable deer using a handheld GPS unit combined with a compass and rangefinder. For much of the year males and females naturally segregate themselves to different sections of the park [19] so they were examined separately. Data on seropositivity (either positive or negative to *T. gondii*) were combined with spatial data by generating heatmaps using the Leaflet [29] and Leaflet.extras [30] packages in R (version 4.1.3) [31]. First, a qualitative approach was used to determine whether the distribution of seropositive versus seronegative deer differed on a large scale (i.e. using heatmaps to determine whether positive individuals were clearly using different areas compared to negative individuals). Second, we calculated Hellinger distances [32]. This produces a metric (ranging from 0 to 1, i.e. from identical distributions to completely different or orthogonal distributions) for assessing the difference in the space use between sexes as well as across serostatus.

In another quantitative approach, we fitted a linear mixed-effect model (*lmer* function of the *lme4* package [33]) to test whether seropositive individuals spent more time closer to buildings in the park. It has been shown in previous studies that due to cats gravitating towards buildings for shelter, that oocyst levels could be higher in areas near them [34,35]. We were interested in testing if this pattern would hold true in this case. As the study site is an urban park, the perimeter is ringed by domestic residences and apartment blocks. There are also a number of buildings inside of the park ranging from the Dublin Zoo (one of Ireland's most well attended tourist attractions), to private residences such as Aras an Uachtaráin (official residence of the president of Ireland) and abandoned buildings (including a magazine fort). Due to the breath of building types included in the analysis, we assumed that approaching buildings would be behaviourally neutral. Bolder individuals may be more likely to approach buildings that are in use in search of food. While more shy individuals may be attracted by the dense undergrowth around abandoned buildings kept in place to shield the buildings from view. An accurate map of buildings in the park was obtained from OSI. We fitted an *a priori* mixed effect model using the following formula:


$$\begin{array}{r} building\;distance\;∼\;toxoplasma\;serostatus+\\ sex+\;season+\;year+\left(1|deer\;ID\right)+Gaussian\;error \end{array}$$

where building distance is a numeric value of the distance in metres from the nearest building for each GPS location where individual deer were recorded during the regular park surveys; toxoplasma serostatus is a categorical variable indicating whether animals tested ‘positive’ or ‘negative’ during the ELISA testing; deer ID is the random intercept denoting the individual identification tag of each individual deer; sex is a categorical variable that distinguishes between ‘males’ and ‘females’; season is a categorical variable indicating the time of the year as the vast majority of males join the female heard during the rut. Season is described as ‘winter/spring’ (January–April), ‘summer’ (May–August), and ‘rut’ (September–December). Finally, year is a categorical variable that differentiates between the different years of location data collection (2018, 2019, 2020, 2021). Year is included to account for any differences in the environment (such as vegetation cover or visitor numbers) that could have occurred in the park during the study period. We include our analysis of potential collinearities in the model in the electronic supplementary materials, S2 [36,37].

### 2.5. Begging ranks: tendency of deer to beg for food *sensu* Griffin *et al*. (2022)

We obtained the begging ranks (on a continuum from deer avoiding any interaction with humans to those persistently seeking contact and begging for food) based on field observations taken over the summers of 2020 and 2021, in the period immediately preceding and simultaneous to our blood data collection. Full data collection protocol and data analysis can be found in Griffin *et al*. [19]. In brief, this was carried out through structured surveys recording as much information as possible on human–deer interactions. Observations included identifying the individual deer involved, the number and identity of deer in the herd at the time, any food being offered, how food was offered, the time of the interaction and the GPS location. These records were used to fit a generalized linear mixed effect model from which best linear unbiased predictor (BLUP) for every individual deer observed. Deer that were observed in less than three observation sessions were excluded from the analysis to reduce the noise created by underrepresented individuals. The base BLUP values were included in our analysis as begging ranks, deer with higher values beg for food more and deer with lower values beg for food less. We also included the uncertainty in the begging ranks in our analysis as it captured the variability overlooked by the singular begging rank value. In general, deer with higher begging ranks have smaller uncertainties as they were observed more frequently than deer with lower begging ranks. These values were computed separately each year as park management altered its approaches for mitigating human–deer interactions. Begging rank (mean: 0.58; first quantile: −0.23; third quantile: 1.49) varied from −2.40 (min value, i.e. the individual avoiding humans the most) to 4.57 (max value, i.e. the individual in the sample population begging for food the most). In general, begging ranks of older individuals tend to be higher than that of younger individuals [19].

### 2.6. Modelling begging rank as a function of seropositivity

Each ELISA reading, expressed as *S*/*P*%, was used as a data point instead of the categorical results of the ELISA kit (seropositive/seronegative). This decision was made in an effort to increase the predictive power of our model. We recorded a large number of equivocal results which could not be included in a categorical model but can be included when examining *S*/*P*%. The ELISA for each sample was run in duplicate, and to be included in the categorical model the results for both findings would have to match. By using *S*/*P*%, we were able to include all the data we collected including both replicates from each tagged animals, giving the model a much better opportunity to take the variability in our ELISA results into account. We differentiated between the two runs in the model using a factor ‘replicate’ which differentiated between run 1 and run 2 of each sample. To these values, we attached the rest of the data such as the individual deer ID, sex, year of collection, begging rank of the individual for that year and the begging rank uncertainty *sensu* Griffin *et al*. [19]. The begging rank uncertainty, in particular, is an important confounding factor which estimates how robust the estimate of the begging rank is based on sample size and number of observations per individual deer [19]. We fitted a linear mixed-effect model to explain the variability of begging rank (response variable representing the willingness of deer to take the risk and approach park visitors) as a function of the *S*/*P*% values, our main predictor, and a set of confounding covariates: sex, begging rank uncertainty, replicate number, year of data collection and age. Type of sample (i.e. filter paper-eluted samples obtained from fawns versus sera collected from culled deer) was omitted from the model equation due to its collinearity with sex [36,37]. Collinearity metrics for this model are available in electronic supplementary materials, S3.

We log-transformed our *S*/*P*% values to improve the model’s fit and to better meet the model’s assumptions of residual normality and homogeneity and we scaled all numeric predictors (i.e. by subtracting the mean and dividing by the s.d.) to improve the model’s convergence. Our model equation was:


$$Begging\;rank\;∼\;\log\left(S/P\%+1\right)+sex+uncertainty\;in\;begging\;rank\;+replicate\;+year\;+age+age^{2}+age^{3}+\left(1|deer\;ID\right)+\;Gaussian\;error$$

where deer ID was fitted as a random intercept in the model, *S*/*P*% and begging rank uncertainty as numerical predictors, and replicate and year of study were fitted as categorical predictors. Age was included with quadratic and cubic effects to allow for nonlinearity (see electronic supplementary material, S4 for more details on why we included the polynomial version of age). For full transparency of model selection, the model was also tested excluding *S*/*P*% and age separately (electronic supplementary material, S5). Linear regression models were created using the lme4 package [33] in R (version 4.1.3) [31]. Data and code used for all analysis are available in the electronic supplementary materials, S6.

## 3. Results

### 3.1. Gondii seropositivity

Over two fawning seasons, we obtained samples from 139 fawns that were tested by ELISA. This was split into 53 fawns in 2020 and 86 fawns in 2021 (seropositivity results for both years in table 1). As the fawns will have obtained their antibodies from their mothers and fallow deer in the Phoenix Park have never been recorded to have more than one offspring per year, we can be confident in reporting the seropositivity rates of all fawns in a single year without including multiple results from the same individual. However, when combining results from both years, it is important that we can identify the mother of each fawn to attribute the results to her. From the fawns we tested, we were able to confidently identify 64 mothers. Seventeen mothers were tested in both fawning seasons with four being removed as their serostatus changed during the study (details below). Of the remaining 60 mothers, 18 (30%) tested positive, 29 (48.3%) tested negative and 13 (21.7%) gave equivocal results. The age of the mothers tested through their fawns spread from a minimum of 2 years to a maximum of 16 years with an average value of 6.4 years.

**Table 1.** Serological screen of blood samples collected from fawns during ear notching and from adult deer during the annual cull. This includes both tagged and untagged individuals unless otherwise stated. Results are based on replicate samples tested using ELISA. Samples with an *S*/*P*% > 50% were considered positive and samples with *S*/*P*% < 40% were considered negative. Samples with *S*/*P*% from 40% to 50% or where the two replicates did not match were considered equivocal. Seroprevalence in females during fawning was estimated by matching individuals fawns with their mothers.

sampling occasion	year(s) of sample collection	*n*	positive	negative	equivocal
fawning	2020	53	13 (24.5%)	34 (64.2%)	6 (11.3%)
fawning	2021	86	24 (27.9%)	33 (38.4%)	29 (33.7%)
combined fawning (Identifiable females only)	2020, 2021	60	18 (30%)	29 (48.3%)	13 (21.7%)
cull	2020	46	5 (10.9%)	37 (80.4%)	4 (8.7%)
cull	2021	42	7 (16.7%)	31 (73.8%)	4 (9.5%)
combined cull	2020, 2021	88	12 (13.6%)	68 (77.3%)	8 (9.1%)

Of the 88 deer sampled during the annual culls, 12 (13.6%) tested positive, 68 (77.3%) tested negative and 8 (9.1%) were equivocal. Seroprevalence of unequivocal samples were similar between the sexes with 12.8% seropositivity in males (5 out of 39 samples) and 17.5% seropositivity in females (7 out of 40 samples) for one animal the sex was not recorded. Nineteen of the culled deer were excluded from further analysis as they had no ear tag, therefore we could not recover any life history information for them regarding begging behaviour or movements within the park. This left us with 61 individuals, 8 of which tested positive (13.1%) with ages ranging from 0 (fawns born approximately 6 months before the cull) to 11 years with an average age of 2.6 years.

Overall 227 samples were tested by ELISA during this study. Seventy-eight of these samples derived from untagged deer. As we cannot track which individual deer these came from we cannot be sure if deer were tested multiple times. The remaining 149 samples came from 125 tagged individuals. Of these tagged individuals, 102 were sampled in only one sampling window (21 testing positive, 66 negative and 15 equivocal). Twenty-two tagged deer were sampled during two sampling windows; 9 tested negative both times, 5 tested negative once and equivocal once, 3 tested positive once and equivocal once, 4 tested negative once and positive once and 1 tested equivocal both times. One individual (Yellow A26) was tested in three sampling windows—twice indirectly (she had a fawn that tested negative in June 2020 and a fawn that tested positive in June 2021), and then again during the cull in November 2021, when she tested negative. All animals that changed their serostatus during the course of the study were excluded from further analysis as it was not possible to determine when exactly they became exposed (and turned seropositive) or cleared the parasite (i.e. turned seronegative). Our overall estimate for seropositivity in this population, based solely on tagged deer, is 20% (24 positive individuals out of 120), with 66.6% negative (80 individuals) and 13.3% equivocal (16 individuals).

### 3.2. Association between spatial behaviour and *T. gondii* serostatus

**Figure 1.**
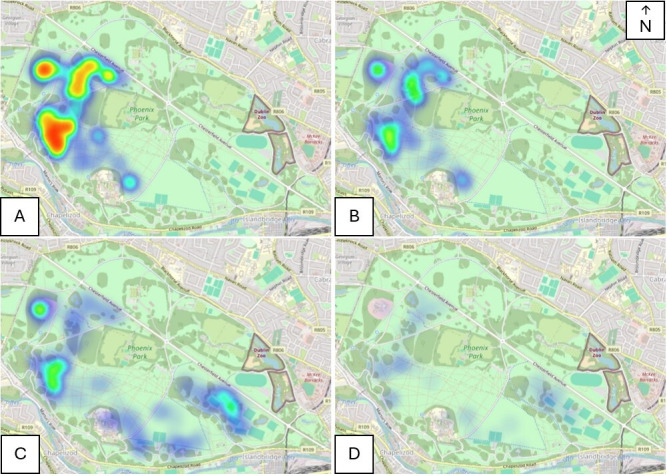
Heatmaps comparing space use by (A) seronegative female, (B) seropositive female, (C) seronegative male and (D) seropositive male deer in Phoenix Park, Dublin.

Figure 1A,B shows heatmaps created to visualize space use by seronegative (*n* = 48) and seropositive (*n* = 21) female deer. There was as strong overlap in space use between the two groups and there were no areas predominantly used by seropositive individuals that were not used by seronegative deer and vice versa. Similar overlaps were obtained for the male deer with seronegative (*n* = 30) and seropositive (*n* = 5) individuals using the same areas of the park (figure 1C,D). Although the relatively small male sample sizes make any interpretation difficult (interactive heatmaps shown in electronic supplementary material, figure S7A,B for females and figure S7C,D for males). From the heatmaps, it is clear that females very seldom use the ‘male area’ to the southeast of the park. However, males often cross over to the ‘female area’, this usually happens in the run up to the rut in the autumn.

We computed the Hellinger distance to evaluate the similarities between deer distributions in the park, where values can range from 0 (identical distributions) to 1 (orthogonal distributions). The Hellinger distance between the sexes (including the rut when sexes are no longer segregated) was calculated as 0.508, whereas the Hellinger distance across serostatus for both sexes was calculated as 0.253, meaning that the degree of separation of animals of different serostatus was lower than the degree of separation driven by sex. Across females the Hellinger distance between seropositive and seronegative individuals was 0.196, indicating almost identical distributions. The same metric was not computed for the males due to low sample size (*n* = 5, seropositive males).

Table 2 provides the results of the mixed effect model explaining the variability of the distance to the buildings. There was no clear link between seropositivity and distance from buildings (see electronic supplementary material, S8 for model effect plots). Males were generally found closer to buildings than females (table 2; electronic supplementary material, S8), whereas both sexes were found to be closer to buildings during the rut (table 2; electronic supplementary material, S8) than during the rest of the year.

**Table 2.** Parameters estimated by the linear mixed effect model explaining the distance from buildings of a deer observed at a point as a function of *T. gondii* status and a range of confounding variables, including sex, season and year. Sex distinguishes between males and females. Serostatus denotes seropositive and seronegative individuals. Season was described as winter/spring (January–April), summer (May–August) and rut (September–December). Year included the four years of observations: 2018, 2019, 2020 and 2021.

predictors	estimates	confidence interval	*t*-value	*p*‐value
intercept	228.24	195.15 – 261.34	13.531	**<0.001**
sex [male]	−38.50	−62.71 – 14.29	−3.096	**0.002**
serostatus [positive]	−3.78	−29.48–21.92	−0.293	0.773
season [summer]	46.57	19.48–73.67	3.325	**0.001**
season [winter/spring]	45.37	20.66–70.09	3.564	**<0.001**
year [2019]	−11.73	−49.79–26.34	−0.589	0.546
year [2020]	−6.85	−46.00–32.30	−0.311	0.731
year [2021]	−35.44	−76.60–5.72	−1.672	0.091
random effects				
*σ* ^2^	17 906.26			
^τ^00 ID	933.20			
ICC	0.05			
*n*_ID_	103			
observations	856			
marginal *R*^2^/conditional *R*^2^	0.036/0.084			

### 3.3. Begging rank as a function of *S*/*P*% ratios

**Figure 2.**
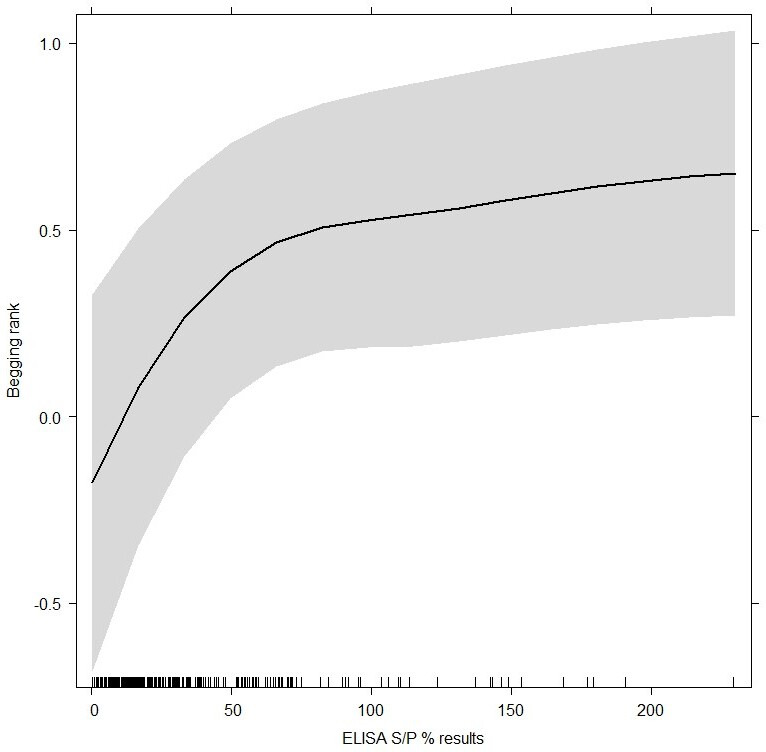
Effect plot depicting the relationship between ELISA *S*/*P*% (*x*-axis) and begging rank (*y*-axis, with greater ranks corresponding to higher likelihood to interact and accept food from park visitors) as predicted by the linear mixed effect model. The shaded area represents the 95% marginal confidence intervals.

**Figure 3.**
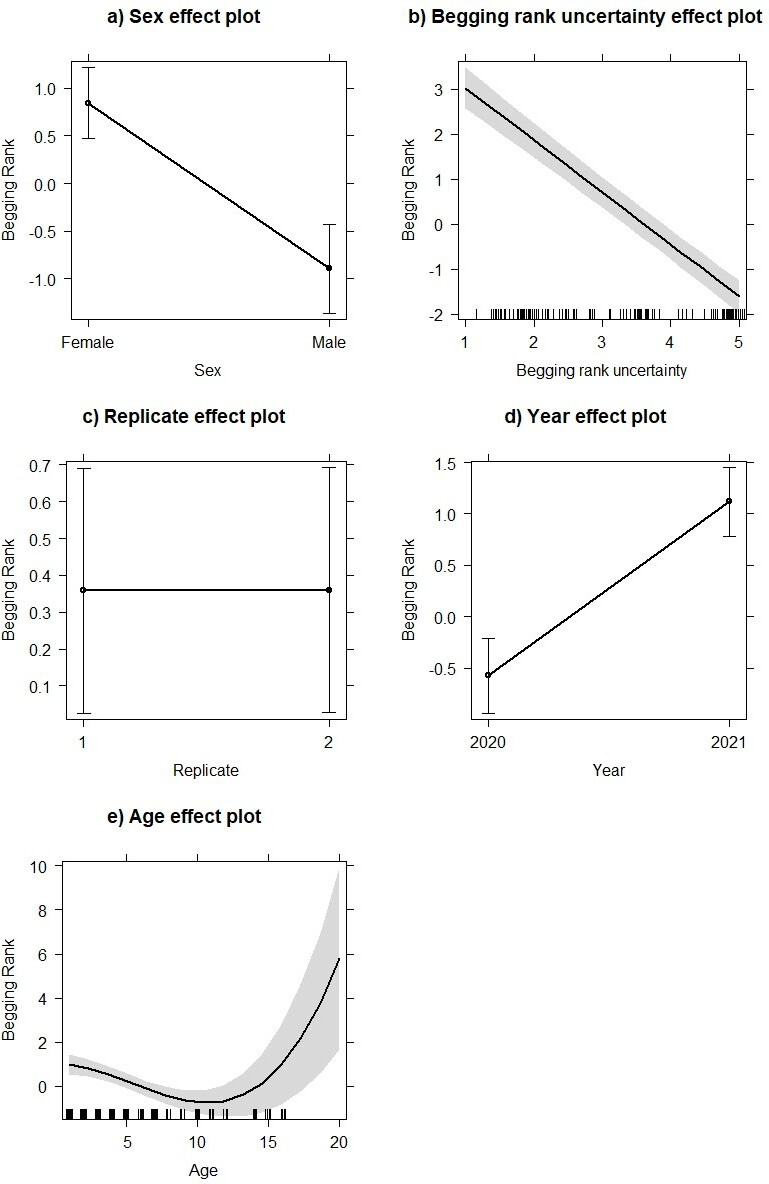
Effect plot depicting the effect of sex (a), begging rank uncertainty (b), replicate test (c), year of study (d) and age (e) on tendency to beg (*y*-axes) in deer monitored in the Phoenix Park, Dublin, as predicted by a linear mixed-effect model. The bars/shaded area represents the 95% marginal confidence intervals.

Results from the mixed-effect model explaining the variability of begging rank as a function of ELISA *S*/*P*% and a range of confounding factors are shown in table 3. The analysis was performed including each ELISA *S*/*P*% result (i.e. including both replicates) rather than the average of the two (thus including a two-level predictor ‘replicate’ as controlling factor). Individuals with higher *S*/*P*% values were associated with an increased tendency to beg for food (table 3, figure 2). Females in our study group were more likely to be beggars (figure 3a) after accounting for begging rank uncertainty, age and sample replicate (figure 3b,c), and in 2021, our cohort had higher begging ranks than the previous year (figure 3d). Consistent with Griffin *et al*. [19], we found that older individuals were more likely to beg (figure 3e).

**Table 3.** Parameters estimated by the linear mixed effect model explaining the variability of begging rank in deer as a function of ELISA *S*/*P*% and a range of confounding variables. The begging rank is a score associated to individual deer based on their likelihood to beg, with higher values meaning more likely to beg. *S*/*P*% are the numeric values obtained from the ELISA test results. Sex is a factor which distinguishes between male and female individuals. Begging rank uncertainty (i.e. the uncertainty arising from the begging rank estimates) ranges from 1.2 to 5.1. Each sample was tested in duplicate and the analysis was performed to include each ELISA S/P% result rather than the average, the factor replicate distinguished between the first and second replicates. The study was run with samples from 2 years (2021 and 2022). Age is the age of each individual in years at the time of sampling and was included with exponents to capture more complicated correlation patterns.

predictors	estimates	confidence interval	*t*-value	*p*‐value
intercept	−0.04	−0.32 – 0.24	−0.270	0.788
*S*/*P%*	0.14	0.05 – 0.24	2.866	**0.005**
begging rank uncertainty	−1.42	−1.57 – 1.28	−19.597	**<0.001**
sex [male]	−1.74	−2.24 – 1.23	−6.760	**<0.001**
replicate [run 2]	0.00	−0.06 – 0.07	0.023	0.982
year [2021]	1.69	1.48 – 1.90	15.839	**<0.001**
age	0.06	−1.63 – 1.75	0.070	0.944
age^2^	−2.77	−6.58 – 1.04	−1.431	0.154
age^3^	2.45	0.16 – 4.73	2.108	**0.036**
random effects				
*σ* ^2^	0.07			
^τ^00 ID	1.39			
ICC	0.95			
*n*_ID_	115			
observations	259			
marginal *R*^2^/conditional *R*^2^	0.451/0.974			

## 4. Discussion

Seroprevalence of *T. gondii* in the fallow deer population in the Phoenix Park differed significantly depending on the timing and cohort of sampling. Samples collected from fawns, which were used to estimate the serostatus of their tagged mothers at the time of fawning, had a seropositivity rate of 30%. As deer are ruminants, mothers pass antibodies to their fawns through their colostrum only during the first few feeds, not across the placenta [38]. Therefore, testing the blood samples of these fawns (which are usually less than 1 week old), can serve as an indirect method to determine whether the mothers are seropositive for *T. gondii*. In contrast, sera from culled deer had a seropositivity rate of just 13.6%. The same difference was observed at repeated sampling occasions (seroprevalence at fawning in 2020 was 24.5% and 27.9% in 2021; seroprevalence at the cull was 10.9% in 2020 and 16.7% in 2021). The age of the mothers of the fawns that were tested was considerably higher (range of 2–16 years, average of 6.4 years) than that of the culled deer (range of 0–11 years, average: 2.6 years) and older animals are generally more likely to be seropositive for *T. gondii* as they have had more opportunity to become exposed over time [39–41]. Another likely explanation (possibly additive to the effect of age) is that periparturient immunosuppression in pregnant females may allow dormant *T. gondii* bradyzoites resident in tissue cysts to reactivate sufficiently to cause an increase in serum antibody levels. Reactivation of chronic infections during pregnancy have been documented in humans [42], and suspected in sheep and goats [43,44]. Moreover, periparturient immune suppression and an associated increase in the shedding of certain parasites have previously been reported for other deer species (red deer [45] and sika deer [46]).

It is important to note that higher *S*/*P*% values are not a definite indicator infection severity [47] and as such we did not expect the behaviour of more highly seropositive individuals to be more severely affected. Although we choose to use the raw values rather than the categorical results, to enable our model to utilize all the information collected, our results neatly coincide with the cut off values recommended in the ELISA kit. We indeed found a sharp change in behaviour predicted by our model between negative (*S*/*P*% < 40) and positive (*S*/*P*%> 50) individuals (figure 2). This contrasts well with the very gentle increase predicted between slightly positive (*S*/*P*% = 50) and very positive (*S*/*P*% = 100) individual (figure 2).

We provided empirical evidence on the fact that the population of fallow deer in Phoenix Park has several *T. gondii* seropositive individuals. The most likely mechanism for herbivorous animals to be infected is through ingestion of oocysts from the faeces of infected cats that have contaminated the environment. One possible route of infection is through water. *T. gondii o*ocysts have been recorded surviving for extended periods of time in freshwater, over 200 days at temperatures routine for our study site [48], allowing them to be carried long distances in watercourses. There is a number of ponds and streams that dot the park, it is possible that deer become infected when interacting with contaminated water sources. Another source of environmental contamination comes from cats burying their faeces. Oocysts can survive for over 100 days in damp soils [49]. As fallow deer primarily graze on grasses they may be likely to ingest soil particulates with their food. It has been reported that rodents caught closer to farm buildings, where there were more cats and consequently more oocysts, are more likely to be infected with *T. gondii* [34,35]. Therefore, before tackling our final and most important question on the association between *T. gondii* serostatus and risk-taking behaviour, we first had to determine whether animals that were naturally less risk averse selected areas of the park where they were less likely to become infected. Contrary to our expectations, there was no correlation between serostatus and distance from buildings. Both qualitative and quantitative analyses showed seropositive and seronegative animals sharing the same areas.

While we feel the most likely mechanism of infection is through contamination of the natural food and water sources by infected cat faeces, there are other potential methods we would like to mention. One method may be through infectious material brought by visitors, such as raw meat containing tissue cysts and/or shoes/clothes contaminated with cat faeces. These risks can be considered very low, but cannot be ruled out. In Ireland relatively low levels of *T. gondii* infections have been reported in livestock [50]. Through previous research in the Phoenix Park, there are occasional records of the deer accepting cooked meat in sandwiches offered by park visitors [19]; however, there have been no observations of visitors feeding deer uncooked meat. As *T. gondii* cannot survive in cooked meat, it would be improbable that any meat fed to the deer would be infected with *T. gondii*. It would also be highly unlikely (but not impossible) that the number of viable oocysts transported via shoe/hand contamination would suffice to infect an immune-competent deer. More research is required, as we could find no reports in the literature examining the transport of oocysts by humans on their hands, clothing or shoes. A final potential pathway of infection is the vertical transmission of *T. gondii* from a mother to surviving offspring. Vertical transmission has been recorded in a number of intermediate host species including sheep [44,51], mice [52] and humans [53]. While there is often negative effects on fetal development, the tolerance for congenital *T. gondii* infection seems to vary by species. However to our knowledge, there are no records of vertical transmission of *T. gondii* in fallow deer specifically so it is difficult to speculate on whether it could be the case here. As we have no empirical evidence on how deer in this population have become exposed to the parasite we can only discuss speculative theories and how they may affect our results. More research is required into how herbivorous species may become infected with *T. gondii* both in peri-urban and rural populations.

The main aim of this study was to verify whether observations of potential host manipulation by *T. gondii* reported rodents under laboratory conditions [2,10,54] translate to free living, non-rodent hosts. In the Phoenix Park, humans are the only major potential threat faced regularly by the deer through annual culls aimed at maintaining a healthy and sustainable population within a walled park. Deer are generally habituated to human presence but avoid any close contact, with the exception of a few individuals that do approach humans [19]. Our analysis showed a significant association between *T. gondii* serostatus and begging behaviour (i.e. the tendency of some individuals to overcome the fear of humans and get closer to take advantage of artificial food items). *T. gondii* antibody levels were indeed positively correlated with begging behaviour, indicating that individuals that had been exposed to *T. gondii* were bolder (less risk averse) than their unexposed counterparts, in line with our main hypothesis on host behavioural manipulation.

Despite this very interesting result, we refrain from claiming that the behaviour of fallow deer is manipulated by *T. gondii*. Our result is novel, and adds important empirical evidence on the dynamics of zoonotic disease maintenance and transmission, but we are not in a position to draw final conclusions about the causal relationship of our result. On the one hand, the likelihood to adopt risk-taking behaviours and approach humans may be boosted by *T. gondii* exposure, along with individual temperament or personality [19,22]. On the other hand, it may be possible that those risk-taking bolder individuals (*sensu* Griffin *et al*. [19]) were more likely to contract the parasite through their contact with humans. Only long-term longitudinal behavioural studies, where deer monitored for years start engaging in risk-taking behaviour after contracting the parasite, will allow us to draw conclusions on the hypothesis of *T. gondii* manipulating its host. From a zoonotic disease perspective, hunters may be more likely to shoot these bolder and/or infected individuals [55,56]. Hunters often handle raw meat without any sort of protection, therefore creating an opportunity for *T. gondii* to pass from one intermediate host to another. Toxoplasmosis has been shown to be widespread across deer populations (see [57]), and our study highlights that humans are likely to interact with seropositive individuals, suggesting that biosecurity measures should be put in place when handling the raw meat of these animals.

## Data Availability

All data and analysis supporting this article are available in the supplementary material [58].
